# Supplementary material for: Application of Host-Depleted Nanopore Metagenomic Sequencing in the Clinical Detection of Pathogens in Pigs and Cats
Source: Animals (Basel). 2023 Dec 13;13(24):3838. doi: 10.3390/ani13243838 (PMC10741237; doi:10.3390/ani13243838)
Supplement: Supplementary file 1 [file animals-13-03838-s001.zip › animals-2723044-supplementary.pdf]

Table S1. PCR Primer Information.

Pathogen	Primer Sequences	Annealing Temperature (°C)	Amplification Product Size (bp)
FPV	F: GTCATACGTACGCTCCTT<br>R: ACTCTTTTACTAACCAAGTCCC	56	1078
FHV-1	F: ACAGACACATATAGGATCAGC<br>R: TAACGTGTATCTGGATTCGTC	57	539
FCV	F: CATGCAATCATACAACCCA<br>R: CACATGTTGATTGGCGGGTA	57	757
FCoV	F: GCTGCACCGTTTATGAGAAG<br>R: ACCAGCTGAAGTTCCAGT	57	726
FIV	F: ATTATGGTGGGGATTTGAA<br>R: ATCTATATACCATGTTTCTGCT	55	712
FeLV	F: CCATGCACACAGACATCCAG<br>R: TATCTGTTGGTACTGTTGGGT	59	443
Mycoplasma felis	F: GTCGGTTTTGTTAACTACGG<br>R: GAGTTTCACCTTCTGTGCTT	55	771
Chlamydia felis	F: TTGTGCTACTTGGTGTGATGCTA<br>R: CAGCGTCGATTAAAGTTGCTC	59	953
Bordetella bronchiseptica	F: CCGCACATTTCCGAACTTCACT<br>R: CCGACAGCACGTCAAAGCC	64	763
CSFV	F: GTGGAGTGGGAGGAATT<br>R: CTCTCACCACTGGAATC	52	989
PRRSV	F: CCAGTCAATCARCTGTGCC<br>R: ATTGAATAGGTGACTYAGAGG	56	420
PCV2	F: GGCCAGTTCGTCACCCTT<br>R: GTAAACTACTCCTCCCGCCAT	56	376
PRV	F: ACGCACGAGGACTACTACGAC<br>R: GTCCATTCGTCACTTCCGGTT	66	241
ASFV	F: GATGCCGATACCACAAGATCAG<br>R: GAGAACGTGAACCTTGCTATTCC	58	869
PEDV	F: TGCCATTCAGCGTATTCTT<br>R: GTAGCCAATACTGCCAGAT	52	925
TGEV	F: GATGGCGACCAGATAGAAG<br>R: TGCAATAGGGTTGCTTGTAC	56	613
PDCoV	F: ACGGTTGGCTGCACTTAATG<br>R: ACTGAGGAGGTGTTGCTGT	56	509
PoRV	F: TATGCTATACCAGTAGGACCA<br>R: GGTCACATCCTCTCACTAT	52	277
16S rRNA	F: AGAGTTTGATCCTGGCTCAG<br>R: TACGGTTACCTTGTTACGACTT	56	1465
STa	F: TGAAAAAGCTAATGTTGGCAAT<br>R: GCAGGATTACAACAAAGTTCACA	56	199

STb	F: ATTTCTTCTTGCATCTATGTTCGT<br>R: CATCCTTTTGCTGCAACCATT	56	195
LT	F: ACGGCGTTACTATCCTGTCTATGTGC<br>R: TTGGTCTCGGTCAGATATGTGATTC	56	275
Stx2e	F: ATCTTCGTTAAATAGTATACGGACA<br>R: CAATTCAGTATAACGGCCACA	56	681
CNF	F: GTTATATAGTCGTCAAGATGGAA<br>R: TCACTAAGCTTTACAATATTGACA	56	636

RNA viruses were subjected to PCR using the QIAGEN OneStep RT-PCR Kit (QIAGEN), and DNA viruses and bacteria were subjected to PCR using the QIAGEN Multiplex PCR Kit (QIAGEN).

Table S2. Sequencing Information for 9 Samples.

Sample No.	Flow Cell and Kit	Host Removal	Host Reads (%)	N50	Generated Reads	Qscore ≥10 fastq.gz files
1	R9.4.1; SQK-LSK110	Yes	45	665b	568.68k	373.5Mb
2	R10.4.1; SQK-NBD114.24	Yes	51	701b	260.6k	240.5Mb
3	R10.4.1; SQK-NBD114.24	Yes	18	548b	884.5k	670.8MB
4	R10.4.1; SQK-NBD114.24	Yes	56	578b	165.4 k	138.1MB
5	R9.4.1; SQK-LSK110	No	77	495b	417.39k	201.8MB
		Yes	67	593b	820.84k	589.4MB
6	R9.4.1; SQK-LSK110	Yes	2	738b	1.33M	630.8MB
7	R9.4.1; SQK-LSK110	Yes	65	560b	466.99k	125.6MB
8	R9.4.1; SQK-LSK110	No	98	1.35kb[1]	559.01k	599.0MB
		Yes	48	687b	819.6k	594.1MB
9	R9.4.1; SQK-LSK110	No	82	518b	2.48M	1.5GB
		Yes	73	556b	2.29M	1.6GB

1. Sample 8, which did not undergo host removal, had a sufficiently high DNA concentration after nucleic acid extraction, eliminating the need for DNA PCR amplification. As a result, the non-amplified DNA sequences were longer, leading to a higher N50 value after sequencing.
